# Benthic communities under anthropogenic pressure show resilience across the Quaternary

**DOI:** 10.1098/rsos.170796

**Published:** 2017-09-20

**Authors:** Julieta C. Martinelli, Luis P. Soto, Jorge González, Marcelo M. Rivadeneira

**Affiliations:** 1 Laboratorio de Paleobiología, Centro de Estudios Avanzados en Zonas Áridas (CEAZA), Av. Bernardo Ossandón 877, CP. 1781681 Coquimbo, Chile; 2 Facultad de Ciencias del Mar, Departamento de Biología Marina, Universidad Católica del Norte, Coquimbo, Chile; 3 Florida Museum of Natural History, University of Florida, Gainesville, FL, USA

**Keywords:** conservation palaeobiology, molluscs, aquaculture, overfishing, temporal baseline, south Pacific

## Abstract

The Southeast Pacific is characterized by rich upwelling systems that have sustained and been impacted by human groups for at least 12 ka. Recent fishing and aquaculture practices have put a strain on productive coastal ecosystems from Tongoy Bay, in north-central Chile. We use a temporal baseline to determine whether potential changes to community structure and composition over time are due to anthropogenic factors, natural climatic variations or both. We compiled a database (*n* = 33 194) with mollusc species abundances from the Mid-Pleistocene, Late Pleistocene, Holocene, dead shell assemblages and live-sampled communities. Species richness was not significantly different, neither were diversity and evenness indices nor rank abundance distributions. There is, however, an increase in relative abundance for the cultured scallop *Argopecten*, while the previously dominant clam *Mulinia* is locally very rare. Results suggest that impacts from both natural and anthropogenic stressors need to be better understood if benthic resources are to be preserved. These findings provide the first Pleistocene temporal baseline for the south Pacific that shows that this highly productive system has had the ability to recover from past alterations, suggesting that if monitoring and management practices continue to be implemented, moderately exploited communities from today have hopes for recovery.

## 1. Introduction

Humans have been exploiting and modifying marine environments around the world for *ca* 160 ka [1–6]. This pressure has increased over the last centuries and decades, leading to fundamental changes in the structure and functioning of these environments [3,4,7,8]. Classical studies incorporating historical, archaeological and palaeontological data have shown that overfishing, in particular, has driven once-abundant large pelagic predators to extinction [5] and has also dramatically altered coastal ecosystems [5,7]. Even if different human groups have been making use of coastal resources for millennia [1,2,9–11], evidence shows that prehistoric human populations did not exert such a strong negative pressure, in comparison to the one marine systems have been suffering in the most recent industrial times [7,10,12,13]. In addition to overfishing, coastal areas are subject to habitat modification, deviation of watercourses, runoff of pollutants, aquaculture, and among others [7,12,14,15]. For example, the damming of the Colorado river caused the collapse of once very abundant populations of the clam *Mulinia coloradoensis,* leading to a dramatic drop in productivity [15], changes to trophic structure [16] and a reduction in carbon emission in the river basin [17]. Ever-growing demands for more food have led to poor aquaculture practices that bring about changes in community structure, ecosystem function, eutrophication and outbreaks of disease [5,18]. Yet, despite considerable efforts to summarize global trends and patterns in exploitation and degradation of coastal areas, most of the available information is limited to the Northern Hemisphere (but see [19] for Tasmania). Our present paradigm is thus lacking information from other coastal environments, also under anthropogenic strain, that can provide insights for a more holistic understanding and potentially a more holistic approach towards remediation and conservation. In this context, coastal marine environments from the Southeast Pacific, where there are highly productive fisheries related to the Humboldt Current System [13,20] are key pieces to add to the puzzle.

**Figure 1.**
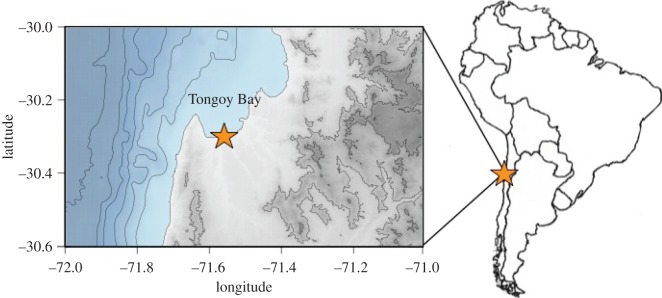
Map of Tongoy Bay on the northern coast of Chile.

The Southeast Pacific is characterized by important upwelling systems along the Chilean and Peruvian coasts [20–22], that have sustained and been impacted by human groups for at least 12 ka [11,23–27]. One of the most productive of these upwellings is located in the north-central region of Chile, near Tongoy (30°12′ S–71°34′ W, [20,21,28], figure 1). Owing to its high productivity this bay has seen the development of small-scale benthic fisheries and of an aquaculture regime of the scallop *Argopecten purpuratus* [29,30]. The uncontrolled exploitation of the scallop since 1945 led to the collapse of this fishery [25,29,30] but, given the economic importance of this resource, an aquaculture regime was implemented in 1988 [29,30]. The area destined for scallop culture is 54% of the surface of Tongoy Bay (1900 out of 3500 ha), and of the remaining 1500 ha, 100 ha are managed by local fishermen as a ‘Management Exploitation Area for Benthic Resources’ (AMERB) [30].

The modern benthic assemblages from Tongoy Bay have been extensively sampled and studied from a trophic network approach [31–34]. These contributions show that the uncontrolled fishing and aquaculture regimes have not only directly impacted the exploited species, but also the structure and functioning of the ecosystem they are a part of [31]. These trophic network studies are incredibly valuable to understand changes to the system in the last 30 years; however, information prior to the 1980s is lacking [31]. In order to assess the magnitude of these changes, it is necessary to use the temporal perspective provided by a Conservation Palaeobiology approach, to compare the current community to a pre-human-impact state [7,15,19,35–42]. Tongoy Bay has dated marine terraces from the Pleistocene and Holocene (HOL) [43–46], making such temporal comparisons feasible.

Here, we seek to use a temporal baseline to contextualize the effect of artisanal fishing and a recent aquaculture regime to a productive coastal ecosystem from north-central Chile. We use palaeontological data from the Pleistocene and Holocene terraces together with recent ecological sampling to quantify community structure and composition over time, to determine whether there are changes and whether these are due to human-related pressures, natural climatic variations or both. We hypothesize that anthropogenic changes have altered community structure and composition over time, and that these will be significantly different in previous ‘states’ of the community. The results from this study will provide one of the first temporal baselines of this kind for the Southern Hemisphere, and in particular for a highly productive upwelling system from the Southeast Pacific.

## 2. Material and methods

### 2.1. Compilation of datasets

We compiled a database using published literature and collected fossil samples from Tongoy Bay. For the Mid-Pleistocene, 22 samples were used (all collected); for the Late Pleistocene, 26 (all collected); for the Holocene, 40 (all collected); for Dead Assemblages, 11 (three collected and eight from the published literature) and for the Live samples, we used a live-collected dataset from [47] and a 2012 live-collected dataset (partly published in [31], table 1). All the fossil samples correspond to bulk-collected unlithified samples comparable to the unlithified Dead Assemblages and the Live samples. Quaternary terraces of Tongoy have been dated by previous studies, showing a strong correlation between height above sea level and age [45,46]; hence, fossil sites were assigned to different interglacial stages from their height above sea level. Species from each time bin were pooled and labelled as follows: Mid-Pleistocene (samples from MIS7 and MIS9 where pooled together and labelled MP), Late Pleistocene, Holocene, Dead Assemblage (DA), Live community (LIVE). For some analyses, we pooled together MP and LP into ‘Pleistocene’ and DA and LIVE into ‘Modern’ (see details below).

**Table 1.** Site details with coordinates, height/depth of collection, estimated age, number of samples and individuals for each time bin. Details of diversity metrics are also presented: species richness, Shannon diversity index and Pielou's *J* evenness index.

site	Lat (South)	Long (West)	height (m)	estimated age	*N* samples	individuals	richness	diversity (*S*)	evenness (*J*)
Tongoy LIVE	whole bay	whole bay	4–25 m deep	Live-collected	591	18 161	36	1.98	0.55
Tongoy DA	30°13′58.8′′	71°28′58.8′′	3–0 m.a.s.l.	Dead Assemblage	11	1934	24	2.19	0.69
Tongoy HOL	30°17′49.2′′	71°32′20.4′′	5 m.a.s.l.	Holocene	40	3242	20	0.87	0.29
Tongoy LP	30°18′0′′	71°34′58.8′′	19–12 m.a.s.l.	Late Pleistocene	26	8189	24	1.38	0.43
Tongoy MP	30°16′33.6′′	71°28′55.2′′	36 m.a.s.l.	Mid-Pleistocene	22	1668	19	1.99	0.67

For the fossil samples, shells from each time bin were identified to species level using published literature [48–51]. After species were identified, bivalve and gastropod individuals were counted. For bivalves, the number of individuals was calculated diving the total number of valves by two. The live-collected data from [47] (table 1) was collected using transects that were laid perpendicular to the bay. Samples were collected by divers using a quadrat at depths between 7 and 25 m. For the second, 2012 study, transects were also laid perpendicular to the bay, and quadrats were used to sample benthic organisms at 4, 8, 12 and 20 m depth. Only the data for bivalves and gastropods were used in order to have consistency with what is preserved in the fossil samples. In addition, the pooled live-collected data and the fossil data were checked for synonymy and updated taxonomy using the WoRMS taxon match online tool (http://www.marinespecies.org/). After the taxonomy was homogenized, we proceeded to separate species into categories depending if they were commercially exploited or non-exploited using the species inventories of the Servicio Nacional de Pesca [52]. The datasets supporting this article have been uploaded as part of the electronic supplementary material and are available on Dryad.

### 2.2. Diversity and abundance metrics

Species relative abundances per time bin (Pleistocene, Holocene and Modern) were calculated to determine whether the older time bin was a good predictor of the one that followed. That is, Pleistocene relative abundance (dependent variable) was regressed on Holocene relative abundance (independent variable), and Holocene relative abundance was regressed on Modern relative abundance. High agreement is indicated by species plotting along a 1 : 1 line in a bivariate plot of relative abundance [53,54]. To test whether the slopes of those regressions were significantly different from 1, we calculated the upper and lower confidence intervals. If a slope of 1 fell within the confidence intervals, we assumed that differences were not significant. The residuals for the regression with the total relative abundances were inspected to identify potential outliers. Species were considered outliers if they had a very high abundance in the dead assemblage and were absent or had low abundances in the living assemblage. For this analysis, we standardized Pleistocene, Holocene and Modern sample numbers by doing a rarefied subsample to the smallest sample number (*n* = 5176, for MP and LP pooled together). The rarefaction was done without replacement, using the ‘rrarefy’ function in the ‘vegan’ package [55], in the statistical programming language R [56].

Diversity indices were calculated for each time bin (MP, LP, HOL, DA and LIVE). For these analyses, we also standardized sample numbers by doing a rarefied subsample (without replacement) to the smallest sample number using ‘rrarefy’. In this case, it was done to the MP sample size (*n* = 1668). We calculated Shannon's diversity index [57] and Pielou's evenness index [58] to determine ‘dead–fossil’ compositional agreement. Indices were calculated with the ‘diversity’ function in the ‘vegan’ package [55] in the statistical programming language R [56].

### 2.3. Similarity metrics for the whole assemblage, exploited and non-exploited species

To test for similarity in species composition between the different time bins (MP, LP, HOL, DA and LIVE) Chao's Jaccard similarity index was used [59]. Chao's Jaccard index includes the effect of species that are shared but unseen (either because they are rare or because the samples that are being compared have substantial differences in size such as these live–dead assemblages). By accounting for unseen species, this estimator is less biased than the classic Jaccard index that is only based on the presence–absence data [59]. The Spearman rank-order correlation of species relative abundance was also used as an indicator of similarity between the different time bins [60]. Chao's Jaccard similarity index and Spearman's rank-order correlation are typically plotted on bivariate plots to represent compositional and abundance similarity between assemblages. In this plot, sites located in the upper right-hand quadrant have the highest agreement and sites in the lower left-hand quadrant have the lowest agreement [53]. Indices were calculated with the ‘diversity’ and ‘chao.jaccard’ functions, in the ‘vegan’ [55] and ‘fossil’ [61] packages in the statistical programming language R [56].

### 2.4. Rank abundance distributions

Species rank abundance plots are also good descriptors of communities [62]. Several theories and models have been proposed to explain the different shape of rank abundance plots in communities (see [62] for a review). Here, we fit three of these models (Geometric series, Broken stick and Zipf) to the rank abundance orders of assemblages from different time bins (Pleistocene, Holocene and Modern) to determine the best-fit model for each dataset. The best model was chosen based on at least a two-point difference in Akaike Information Criterion (AIC). For this analysis, we also standardized Pleistocene, Holocene and Modern sample numbers by doing a rarefied subsample without replacement to *n* = 5176. We carried out these analyses with the ‘rrarefy’ and ‘fitrad’ functions in the ‘vegan’ [55] and ‘sads’ [63] packages in the statistical programming language R [56].

### 2.5. Abundance and body size of *Argopecten* and *Mulinia* through time

The relative abundance (i.e. proportion of the total individuals) of the scallop *Argopecten purpuratus* (Lamarck 1819) and the clam *Mulinia edulis* (King & Broderip 1831) were quantified per time bin. These two species were selected as they were very abundant in fossil samples, and are subject to fishing pressure and aquaculture (only *Argopecten*) nowadays.

Changes in body size can be indicative of subsistence harvesting [64] and fishing pressure [65]. Species typically exhibit decreasing body size, indicative of an overexploitation of larger size classes [64,65]. The two most abundant species in Tongoy Bay are subject to different anthropogenic pressures as *Argopecten* is cultivated, but *Mulinia* is not. Thus, it is possible there are variations in size between the fossil and the dead samples for these species. To explore this, specimens from *Argopecten* (*n* = 135) and *Mulinia* (*n* = 3803) were measured to the nearest millimetre using a digital caliper, and their body size was calculated per time bin as the geometric mean of shell height and shell length [66].

## 3. Results

### 3.1. Diversity and abundance metrics

The samples collected and compiled from the literature (*n* = 690, table 1) yielded a live assemblage with 18 161 individuals from 36 species, DAs with 1934 shells from 24 species, and fossil assemblages with 3242 individuals and 20 species from the HOL, 8189 and 24 from the LP, and 1668 and 19 from the MP (table 1). The combined richness was of 62 species.

Univariate raw unstandardized diversity metrics are not different between time periods (table 1). Species richness is not significantly different (Kruskal–Wallis rank sum test, *χ*^2^ = 4, *p* = 0.406), neither are Shannon's or Pielou's diversity and evenness indices (Kruskal–Wallis rank sum test, *χ*^2^ = 4, *p* = 0.406 for both). When Pleistocene and Holocene samples are pooled together, linear models indicate that abundances of Pleistocene species are significant predictors of the Holocene species relative abundance (adjusted *R*^2^ = 0.20, *F* = 5.83, *p* = 0.03, electronic supplementary material, figure S1*a*); however, pooled Holocene species are not significant predictors of Modern species relative abundances (adjusted *R*^2^ = −0.05, *F* = 0.009, *p* = 0.92; electronic supplementary material, figure S1*b*), suggesting a shift in species composition and/or relative abundances in the live-collected samples. Confidence intervals indicate that slopes are slightly lower than 1 for the Pleistocene and Holocene (lower CI = 0.07, upper CI = 0.96), but the Holocene and Modern slope is significantly lower than 1 (lower CI = −0.48, upper CI = 0.53).

### 3.2. Similarity metrics for the whole assemblage, exploited and non-exploited species

**Figure 2.**
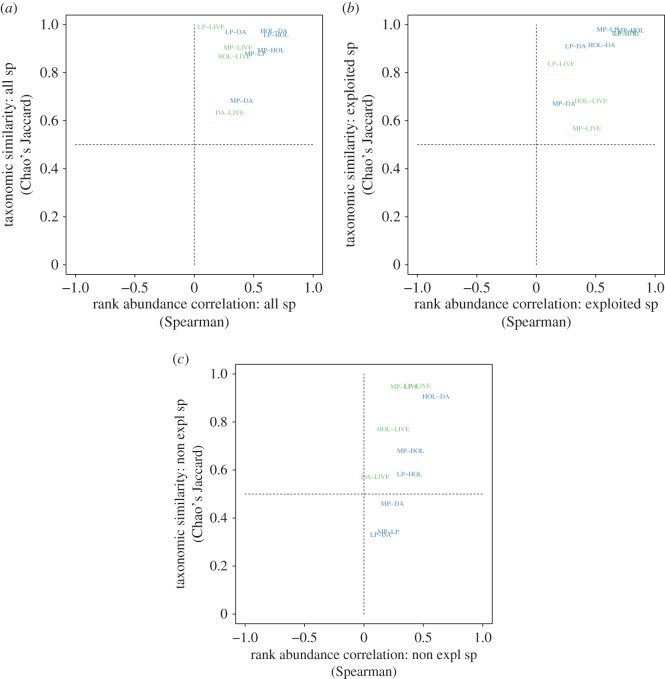
Bivariate plots of taxonomic similarity (Chao's Jaccard) and rank-order correlation of relative abundances (Spearman's *ρ*) for temporal assemblages from Tongoy Bay, Chile. Each point represents a comparison between a different time for the same site, i.e. MP-LP compares Mid-Pleistocene and Late Pleistocene assemblages from Tongoy Bay. Sites located in the upper right-hand quadrant in each panel have the highest live–dead/fossil agreement. All the comparisons between fossil assemblages are depicted in blue whereas comparisons with the Live assemblages are depicted in green. (*a*–*c*) The results for analyses with (*a*) all species, (*b*) only exploited species and (*c*) only non-exploited species.

Chao's Jaccard index for assemblage-level compositional similarity between time periods is higher than 0.64 for all comparisons (electronic supplementary material, table S1; figure 2*a*). This similarity between samples suggests stability in composition throughout the span of the Quaternary analysed. A visual inspection of the bivariate plot with Chao's Jaccard similarity index and Spearman's *ρ* (figure 2*a*) shows that the samples fall in the upper right-hand quadrant, indicating that live–dead agreement is high [53]. Significance for Spearman's rank correlations between samples is shown in the electronic supplementary material, table S1.

When species were subdivided into ‘Exploited’ and ‘Non-exploited’ categories it became evident that ‘Exploited’ species were driving the dissimilarity between live-collected and fossil samples (electronic supplementary material, table S1; figure 2*b*,*c*). The species classified as ‘Exploited’ (or ‘exploitable’ in the case of the fossil samples that are prior to documented human exploitation) make up a large proportion of the community. Moreover, ‘Non-exploited’ species tend to be less abundant, sometimes rare. Thus, it is not surprising that the data show a less clear pattern for the latter (figure 2*b*,*c*). Spearman's *ρ* is significant for only half of the comparisons in ‘Exploited’ and ‘Non-exploited’ (electronic supplementary material, table S1). A visual inspection of figure 2*b*,*c* shows that comparisons between fossils and the live-collected samples plot in the upper right-hand quadrant for ‘Exploited’, but differ for just ‘Non-exploited’.

### 3.3. Rank abundance distributions

**Figure 3.**
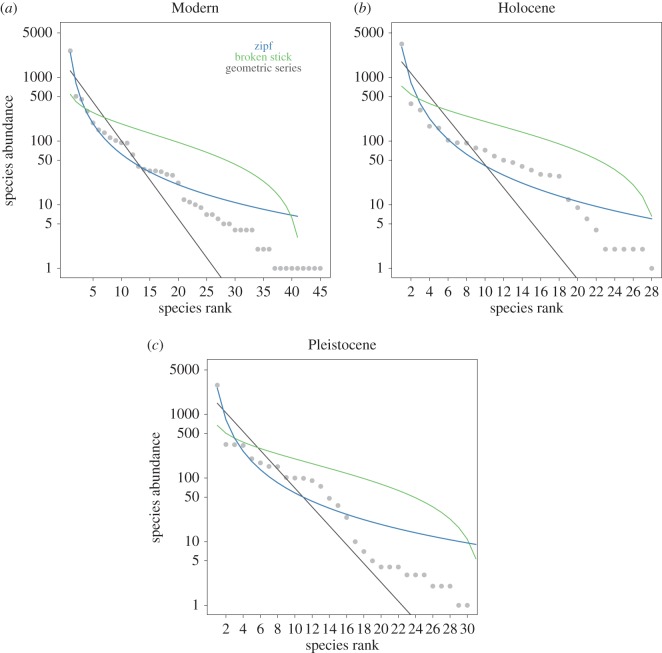
Rank abundance distribution for (*a*) Modern, (*b*) Holocene and (*c*) Pleistocene assemblages. Fossil assemblages were resampled to total Modern assemblage abundance (*n* = 5176). The lines for fits of three rank abundance distribution models are overlaid, where Zipf is the best-fitting model for all time periods (electronic supplementary material, table S2).

Species rank abundance distributions for Modern, Holocene and Pleistocene assemblages are best explained by the same model, as indicated by AIC (electronic supplementary material, table S2; figure 3*a*–*c*). The model with the strongest support is Zipf.

### 3.4. Abundance of *Argopecten* and *Mulinia* through time

**Figure 4.**
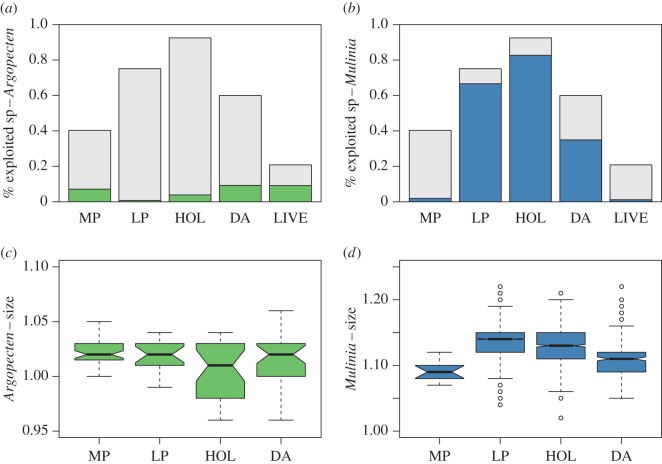
These figures show changes through time in relative abundance (*a*,*b*) and body size (*c*,*d*) for *Argopecten purpuratus* (in green) and *Mulinia edulis* (in blue). Percentage of exploited species per time bin is shown in panels *a*,*b* (light grey), overlaid is the proportion of these exploited species that corresponds to (*a*) *Argopecten* and (*b*) *Mulinia.* (*c*,*d*) Body size through time for (*c*) *Argopecten* and (*d*) *Mulinia*. Horizontal bars represent median values, boxes enclose the 25th to 75th percentiles, whiskers indicate the minimum and maximum values and the dots are outliers.

The relative abundance of ‘Exploited’ and ‘Non-exploited’ species was calculated for each time bin (figure 4*a*,*b*). For the LIVE assemblage 21% of the individuals belong to exploited species and 50% of that corresponds to the scallop *Argopecten*. If these values are compared to those from the older fossil assemblages, the individuals that belong to exploited species make up between 40% (MP) and 92% (HOL) but *Argopecten* was responsible for less than a fifth of this, and the clam *Mulinia* for over 50% (figure 4*b*). Thus, locally harvested *Argopecten* shows an increase in relative abundance in LIVE compared with fossil assemblages, whereas *Mulinia* used to be very abundant in the recent past (over 80% of the ‘exploitable’ species) but has decreased in abundance and is rare in the LIVE assemblage (figure 4*b*).

### 3.5. Body size of *Argopecten* and *Mulinia* through time

The clam *Mulinia* shows a significant decrease in body size from the HOL to the DA as well as between MP and all the other time bins (electronic supplementary material, table S3; figure 4*d*). Unfortunately, there is no comparable size data for live-collected *Mulinia*, but using data from Stotz *et al*. [67] we determined that for *Mulinia* from a neighbouring bay, the mean length is 37 mm while the mean length for the HOL and DAs from the samples used here are 53 and 52 mm, respectively (see the electronic supplementary material datasets). Thus, there has been a decrease in mean length of 15–16 mm. The body size of the scallop *Argopecten*, in contrast, shows less variability through time (figure 4*c*), with only MP shells showing significant differences in size with HOL shells (electronic supplementary material, table S3).

## 4. Discussion

Assemblage-level metrics of diversity and compositional similarity over time suggest that the benthic community at Tongoy Bay has remained relatively stable through the Quaternary. Moreover, despite fishing and aquaculture pressure, in the last two decades the benthic community shows signs of resilience and recovery [31]. When results are considered for two of the most abundant species, however, this general positive outlook becomes questionable. There is an artificial increase in the relative abundance of *Argopecten*, while *Mulinia* is nowadays very rare, and only consistently found alive in neighbouring bays. The increase in *Argopecten* abundance is probably a reflection of aquaculture practices, yet we are unaware of any detrimental effects this practice could have had on *Mulinia*. The absence of this clam may be due to natural environmental changes in the region, local changes in freshwater input to the bay (see ‘Reconstructing the regional history of *Mulinia*’ in the Discussion), and/or to more recent anthropogenic impacts such as disease by parasites (see ‘Recent local anthropogenic pressures on the benthic community’ in the Discussion). Results indicate that natural and anthropogenic stressors probably impacted the populations at different times, with strong anthropogenic impacts being quite recent (less than 50 years). Findings from this study suggest that benthic communities from the south Pacific show strong resilience and recovery potential, but drivers of change and their impacts on key species need to be better understood if benthic resources are to be preserved in the near future. Moreover, even if it exceeds the scope of the manuscript, it is worth emphasizing that in order to reconstruct changes in the community over time a Conservation Palaeobiology approach is fundamental. This would require doing more detailed palaeoenvironmental reconstructions together with a refined stratigraphic framework.

### 4.1. Stability and recovery despite human pressure

The stability or resilience suggested by the mollusc data from Tongoy Bay is in agreement with global studies that also indicate high recovery potential in marine ecosystems [7,68]. For example, fossil marine faunas have shown stasis through time [69–71] while modern marine faunas seem to have high recovery potential, returning to conditions similar to the original one in decades [7,72,73]. More specifically for molluscs, it has been shown that the composition of bivalves and gastropods from reefs in the Bahamas had remained stable from the Pleistocene [74]. Other studies have nevertheless found remarkable differences between marine communities from the Pleistocene and Holocene with present-day assemblages [39,70]. For example, fossil molluscs from core samples collected in the Adriatic Sea had different composition, diversity and dominance than their recent counterparts, suggesting these communities remained relatively unchanged throughout glacial–interglacial cycles but shifted in composition in recent times probably due to anthropogenic impacts [39]. Similarly, coral communities from Barbados showed resilience and stability through the Pleistocene but dramatic shifts in composition and abundance were observed in recent coral species relative to fossil ones [70]. Results from these temporal studies can, however, be contingent on the scale of analyses [75]. Similar research on molluscs found that species composition was highly similar between Pleistocene and Modern samples when viewed at a regional scale, but similarity decreased when comparisons were done at a local scale [75]. In addition, preliminary results suggest strong differences in species composition between Modern and Pleistocene assemblages in other localities in north-central Chile [42].

### 4.2. Reconstructing the regional history of *Mulinia*

Despite the stability in composition and abundance in the mollusc community, and the lack of significant differences between the measured indices, *Mulinia* is locally very rare in Tongoy Bay and is only consistently found alive in neighbouring bays [67]. This marked change in the dominance of the clam can be a consequence of natural and anthropogenic causes. Results at hand, together with regional information, previous studies on congeneric species, and fisheries landings suggest that the disappearance of the clam was mainly due to natural factors. At a regional level, *Mulinia* was the most common bivalve in Pleistocene assemblages from northern Chile and Peru [44]. There are, however, notable differences in abundance and size for *Mulinia* between the MP and LP. This could be associated with the much higher uplift rates estimated for Tongoy Bay during the MP (in particular MIS 7 and MIS 9) compared with the LP [45]. These high uplift rates induced by tectonic processes could have led to a higher probability of coastal modification and habitat destruction, which could have impacted molluscan abundance and size. The species seems to have recovered in the LP. During the Holocene, however, the record of the *Mulinia* is very sparse, as the clam practically disappeared from northern Chile and is only found in one Holocene terrace in Peru (Michilla terrace around 7 ka [44]). Thus, it has been suggested that the dramatic shrinking of the species distribution occurred in the Holocene, possibly by a combination of palaeoceanographic circumstances together with biological phenomena [44]. More recent palaeoclimatic studies in northern Chile and Peru support this idea, given that aridity increased in the region during the Mid-Holocene [76–79]. Specific dates vary but most studies indicate that a displacement of the southern Westerlies (which control an N-S precipitation gradient) caused arid conditions between 7.7 and 4.2 ka [76–79]. This aridity could have indirectly affected *Mulinia* due to a decrease in freshwater input.

Previous studies show that populations of the congeneric *Mulinia coloradoensis* from the Northern Hemisphere were decimated by a decrease in freshwater and nutrient input [15]. These changes caused by the damming of the Colorado river significantly decreased the productivity of the associated estuary and brought about detrimental ecosystem-level consequences [15–17,80]. Therefore, it is likely that *Mulinia* from Chile and Peru could have also thrived in estuary conditions during more humid weather. This idea is further supported by the presence of the species in estuaries in Los Lagos region, southern Chile.

Another local line of evidence suggesting that a freshwater input is important for the species comes from recent fisheries landings in the area (electronic supplementary material, figure S2). According to this information, the species was initially not exploited (first records are from 1994) due to low abundance but there was a boost in the population during 1997, a strong ENSO year [20], and a sharp decline in landings afterwards. The increase in rain that year brought water to previously dry riverbanks that discharge in Tongoy Bay, perhaps creating favourable conditions for the clam and leading to an increased catchment by the local fishermen towards the end of the year. Regardless of this isolated local event, if we consider together the evidence from regional climate changes and life-history information of congeneric species, we can suggest that the disappearance of the clam *Mulinia* was probably a regional phenomenon driven by natural changes in environmental conditions, rather than a consequence of negative human impacts.

### 4.3. Recent local anthropogenic pressures on the benthic community

Despite the strong evidence pointing to natural regional changes, human-induced aquaculture and fishing pressures might also be having a more recent detrimental effect. Studies looking at the status of the system in Tongoy Bay from a trophic network approach found that the overall health of the community improved from 1994 to 2012 [31]. This apparent recovery was brought about by release from fishing pressure due to the establishment of better management practices in the 1990s. *Mulinia* is, however, still not present in the living community, and the banks of the species in neighbouring bays are 97% infected with a trematode parasite that attacks soft tissue, the gills in particular [67]. No information is yet available on the parasite species or where it came from but its presence might be related to aquaculture practices in the bay [81]. For example, the scallop *Argopecten* has numerous described parasites and commensals [82] that could also use *Mulinia* as a host. Therefore, even if the major changes to species populations and abundance over time appear to have been natural, there are anthropogenic factors that presently pose a threat, might have not left a record yet and should thus continue to be closely monitored.

### 4.4. Caveats and closing remarks

So far, results indicate that the benthic environment is Tongoy Bay has been stable through the Quaternary and if changes occurred, the system showed resilience. Nevertheless, a caveat to consider is that this study was centred on the benthic mollusc community, limiting our understanding of large and/or pelagic organisms, for example. Previous research [3,5,83] has shown that large pelagic predators were decimated in tropical seas worldwide, leading to changes in functionality in the whole community. We are unaware if something like this happened to large pelagic fish in Tongoy Bay but the evidence at hand shows that mollusc communities are good surrogates for the benthic community [84], suggesting that our findings are at least a reliable representation of changes to this part of the community over time.

Here, we present what is to our knowledge the first Conservation Palaeobiology study for South America and for a highly productive upwelling system in the south Pacific Ocean. Our results suggest that the marine benthic community shows resilience and recovery from the Mid-Pleistocene onwards. An important decrease in relative abundance to the once dominant clam *Mulinia* seems to respond to natural climatic shifts in the Holocene. There are, however, indications that recent anthropogenic pressures may lead to unseen changes in the region as the clam is infested by parasites in neighbouring bays, and the scallop *Argopecten* has an artificially increased relative abundance probably due to aquaculture. These findings provide a temporal baseline that shows that this highly productive system has had the ability to recover from past alterations. Nonetheless, trophic network studies in Tongoy Bay [31] and in central Chile [85] suggest that fisheries can heavily modulate subtidal communities, with impacts extending even to non-harvested species [85]. Therefore, whether more recent human pressures will modify this long-time history of resilience remains to be seen. This temporal perspective provides an understanding of the variability displayed by this benthic marine system in the past, which is critical in order to manage for the future [36,37,86,87]. Studies of this kind are much needed to better comprehend recent changes to global marine communities where the literature is dominated by examples from the Northern Hemisphere and tropical environments. Assuming the system continues to behave as in the past, findings presented here suggest that if monitoring and management practices continue to be implemented, the moderately exploited communities from today have high hopes for recovery.

## Supplementary Material

Supplementary material for Martinelli et al.

## Data accessibility

Supporting data are available as supplementary online material (data, figures, tables and R code). The data are available on Dryad: http://dx.doi.org/10.5061/dryad.2g5f9 [88].

## References

[1] Erlandson JM, Rick TC 2010 Archaeology meets marine ecology: the antiquity of maritime cultures and human impacts on marine fisheries and ecosystems. Annu. Rev. Mar. Sci. 2, 231–251. (doi:10.1146/annurev.marine.010908.163749) 10.1146/annurev.marine.010908.163749 21141664

[2] Griffiths CL et al. 2005 Impacts of human activities on marine animal life in the Benguela: a historical overview. Oceanogr. Mar. Biol. Annu. Rev. 42, 303–392. (doi:10.1201/9780203507810.ch8)

[3] Jackson JB 2001 What was natural in the coastal oceans? Proc. Natl Acad. Sci. USA 98, 5411–5418. (doi:10.1073/pnas.091092898) 11344287 10.1073/pnas.091092898 PMC33227

[4] Jackson JB 2008 Ecological extinction and evolution in the brave new ocean. Proc. Natl Acad. Sci. USA 105, 11 458–11 465. (doi:10.1073/pnas.0802812105) 18695220 10.1073/pnas.0802812105 PMC2556419

[5] Jackson JB et al. 2001 Historical overfishing and the recent collapse of coastal ecosystems. Science 293, 629–637. (doi:10.1126/science.1059199) 11474098 10.1126/science.1059199

[6] Marean CW et al. 2007 Early human use of marine resources and pigment in South Africa during the Middle Pleistocene. Nature 449, 905–908. (doi:10.1038/nature06204) 17943129 10.1038/nature06204

[7] Lotze HK et al. 2006 Depletion, degradation, and recovery potential of estuaries and coastal seas. Science 312, 1806–1809. (doi:10.1126/science.1128035) 16794081 10.1126/science.1128035

[8] Worm B et al. 2006 Impacts of biodiversity loss on ocean ecosystem services. Science 314, 787–790. (doi:10.1126/science.1132294) 17082450 10.1126/science.1132294

[9] Báez P, Jackson Squella D 2008 Exploitation of loco, *Concholepas concholepas* (Gastropoda: Muricidae), during the Holocene of Norte Semiarido. In *Early Human Impacts on Megamolluscs* (eds AT Antczak, R Cipriani), pp. 79–94. Oxford, UK: Archaeopress Publishers of British Archaeological Reports.

[10] Erlandson JM, Rick TC, Braje TJ, Steinberg A, Vellanoweth RL 2008 Human impacts on ancient shellfish: a 10,000 year record from San Miguel Island, California. J. Archaeol. Sci. 35, 2144–2152. (doi:10.1016/j.jas.2008.01.014)

[11] Jerardino A, Castilla JC, Ramirez JM, Hermosilla N 1992 Early coastal subsistence patterns in central Chile: a systematic study of the marine-invertebrate fauna from the site of Curaumilla-1. Lat. Am. Antiquity 3, 43–62. (doi:10.2307/971929)

[12] Halpern BS et al. 2008 A global map of human impact on marine ecosystems. Science 319, 948–952. (doi:10.1126/science.1149345) 18276889 10.1126/science.1149345

[13] Pauly D, Christensen V, Dalsgaard J, Froese R, Torres F 1998 Fishing down marine food webs. Science 279, 860–863. (doi:10.1126/science.279.5352.860) 9452385 10.1126/science.279.5352.860

[14] Casey MM, Dietl GP, Post DM, Briggs DE 2014 The impact of eutrophication and commercial fishing on molluscan communities in Long Island Sound, USA. Biol. Conserv. 170, 137–144. (doi:10.1016/j.biocon.2013.12.037)

[15] Kowalewski M, Serrano GEA, Flessa KW, Goodfriend GA 2000 Dead delta's former productivity: two trillion shells at the mouth of the Colorado River. Geology 28, 1059–1062. (doi:10.1130/0091-7613(2000)28<1059:DDFPTT>2.0.CO;2)

[16] Cintra-Buenrostro CE, Flessa KW, Avila-Serrano G 2005 Who cares about a vanishing clam? Trophic importance of *Mulinia coloradoensis* inferred from predatory damage. Palaios 20, 296–302. (doi:10.2110/palo.2004.p04-21)

[17] Smith JA, Auerbach DA, Flessa KW, Flecker AS, Dietl GP 2016 Fossil clam shells reveal unintended carbon cycling consequences of Colorado River management. R. Soc. open sci. 3, 160170 (doi:10.1098/rsos.160170) 27703685 10.1098/rsos.160170 PMC5043302

[18] Naylor RL et al. 2000 Effect of aquaculture on world fish supplies. Nature 405, 1017–1024. (doi:10.1038/35016500) 10890435 10.1038/35016500

[19] Edgar G, Samson C 2004 Catastrophic decline in mollusc diversity in eastern Tasmania and its concurrence with shellfish fisheries. Conserv. Biol. 18, 1579–1588. (doi:10.1111/j.1523-1739.2004.00191.x)

[20] Thiel M et al. 2007 The Humboldt Current System of Northern and Central Chile: oceanographic processes, ecological interactions and socioeconomic feedback. Oceanogr. Mar. Biol. 45, 195–344. (doi:10.1201/9781420050943.ch6)

[21] Daneri G, Dellarossa V, Quiñones R, Jacob B, Montero P, Ulloa O 2000 Primary production and community respiration in the Humboldt Current System off Chile and associated oceanic areas. Mar. Ecol. Prog. Ser. 197, 41–49. (doi:10.3354/meps197041)

[22] Riascos JM, Uribe RA, Donayre S, Flores D, Galindo O, Quispe C, Gonzalez J 2016 Human footprints on benthic communities in marine reserves: a study case in the most productive upwelling system worldwide. Mar. Ecol. Prog. Ser. 557, 65–75. (doi:10.3354/meps11857)

[23] Marquet PA, Santoro CM, Latorre C, Standen VG, Abades SR, Rivadeneira MM, Arriaza B, Hochberg ME 2012 Emergence of social complexity among coastal hunter-gatherers in the Atacama Desert of northern Chile. Proc. Natl Acad. Sci. USA 109, 14 754–14 760. (doi:10.1073/pnas.1116724109) 10.1073/pnas.1116724109 PMC3443180 22891345

[24] Sandweiss DH 2003 Terminal Pleistocene through Mid-Holocene archaeological sites as paleoclimatic archives for the Peruvian coast. Palaeogeogr. Palaeoclimatol. Palaeoecol. 194, 23–40. (doi:10.1016/s0031-0182(03)00270-0)

[25] Santoro C et al. 2016 Cazadores, recolectores y pescadores arcaicos del Desierto de Atacama. entre el Pacífico y los Andes, norte de Chile (ca. 10.000–3.700 años ap). Santiago, Chile: Prehistoria en Chile desde sus Primeros Habitantes hasta los Incas. Editorial Universitaria.

[26] Santoro CM, Rivadeneira MM, Latorre C, Rothhammer F, Standen VG 2012 Rise and decline of chinchorro sacred landscapes along the hyperarid coast of the Atacama Desert/Auge Y Decadencia Del Paisaje Sagrado De Los Chinchorro En La Costa Hiperárida Del Desierto De Atacama. Chungara 44, 637–653. (doi:10.4067/S0717-73562012000400007).

[27] Santoro CM, Arriaza BT, Standen VG, Marquet PA 2005 People of the Coastal Atacama Desert: Living Between Sand Dunes and Waves of the Pacific Ocean. In *Desert Peoples: Archaeological Perspectives* (eds P Veth, M Smith, P Hiscock), pp. 243–260. Wiley Online Library. (doi:10.1002/9780470774632.ch13)

[28] Fonseca T, Farías M 1987 Estudio del proceso de surgencia en la costa chilena utilizando percepción remota. Investig. Pesqueras 34, 33–46.

[29] Stotz W 2000 When aquaculture restores and replaces an overfished stock: is the conservation of the species assured? The case of the scallop *Argopecten purpuratus* in northern Chile. Aquacult. Int. 8, 237–247. (doi:10.1023/A:1009215119051)

[30] Stotz WB, González SA 1997 Abundance, growth, and production of the sea scallop *Argopecten purpuratus* (Lamarck 1819): bases for sustainable exploitation of natural scallop beds in north-central Chile. Fish. Res. 32, 173–183. (doi:10.1016/S0165-7836(97)00010-6)

[31] González J, Ortiz M, Rodríguez-Zaragoza F, Ulanowicz RE 2016 Assessment of long-term changes of ecosystem indexes in Tongoy Bay (SE Pacific coast): based on trophic network analysis. Ecol. Indic. 69, 390–399. (doi:10.1016/j.ecolind.2016.04.019)

[32] Ortiz M, Wolff M 2002 Trophic models of four benthic communities in Tongoy Bay (Chile): comparative analysis and preliminary assessment of management strategies. J. Exp. Mar. Biol. Ecol. 268, 205–235. (doi:10.1016/S0022-0981(01)00385-9)

[33] Ortiz M, Wolff M 2002 Spatially explicit trophic modelling of a harvested benthic ecosystem in Tongoy Bay (central northern Chile). Aquat. Conserv. 12, 601–618. (doi:10.1002/aqc.512)

[34] Wolff M 1994 A trophic model for Tongoy Bay—a system exposed to suspended scallop culture (Northern Chile). J. Exp. Mar. Biol. Ecol. 182, 149–168. (doi:10.1016/0022-0981(94)90048-5)

[35] Cramer KL, Leonard-Pingel JS, Rodriguez F, Jackson JB 2015 Molluscan subfossil assemblages reveal the long-term deterioration of coral reef environments in Caribbean Panama. Mar. Pollut. Bull. 96, 176–187. (doi:10.1016/j.marpolbul.2015.05.031) 26031382 10.1016/j.marpolbul.2015.05.031

[36] Dietl GP, Flessa KW 2011 Conservation paleobiology: putting the dead to work. Trends Ecol. Evol. 26, 30–37. (doi:10.1016/j.tree.2010.09.010) 21035892 10.1016/j.tree.2010.09.010

[37] Dietl GP, Kidwell SM, Brenner M, Burney DA, Flessa KW, Jackson ST, Koch PL 2015 Conservation paleobiology: leveraging knowledge of the past to inform conservation and restoration. Annu. Rev. Earth Planet. Sci. 43, 79–103. (doi:10.1146/annurev-earth-040610-133349)

[38] Kosnik MA, Kowalewski M 2016 Understanding modern extinctions in marine ecosystems: the role of palaeoecological data. Biol. Lett. 12, 20150951 (doi:10.1098/rsbl.2015.0951) 27048464 10.1098/rsbl.2015.0951 PMC4881335

[39] Kowalewski M, Wittmer JM, Dexter TA, Amorosi A, Scarponi D 2015 Differential responses of marine communities to natural and anthropogenic changes. Proc. R. Soc. B 282, 20142990 (doi:10.1098/rspb.2014.2990) 10.1098/rspb.2014.2990 PMC4345463 25673689

[40] Pauly D 1995 Anecdotes and the shifting baseline syndrome of fisheries. Trends Ecol. Evol. 10, 430 (doi:10.1016/S0169-5347(00)89171-5) 21237093 10.1016/s0169-5347(00)89171-5

[41] Rick TC, Lockwood R 2013 Integrating paleobiology, archeology, and history to inform biological conservation. Conserv. Biol. 27, 45–54. (doi:10.1111/j.1523-1739.2012.01920.x) 22979917 10.1111/j.1523-1739.2012.01920.x

[42] Rivadeneira MM, Santoro CM, Marquet PA 2010 Reconstructing the history of human impacts on coastal biodiversity in Chile: constraints and opportunities. Aquat. Conserv. 20, 74–82. (doi:10.1002/aqc.1051)

[43] Ota Y, Miyauchi T, Paskoff R, Koba M 1995 Plio-Quaternary marine terraces and their deformation along the Altos de Talinay, north-central Chile. Andean Geol. 22, 89–102. (doi:10.5027/andgeoV22n1-a05)

[44] Paskoff R, Leonard E, Novoa J, Ortlieb L, Radtke U, Wehmiller J 1995 Field meeting in the La Serena-Coquimbo Bay area (Chile). Guidebook for a fieldtrip. In Annual Meeting of the International Geological Correlation Program (IGCP) Project 367, Antofagasta, Chile, 19–28 November.

[45] Saillard M, Hall S, Audin L, Farber D, Hérail G, Martinod J, Regard V, Finkel R, Bondoux F 2009 Non-steady long-term uplift rates and Pleistocene marine terrace development along the Andean margin of Chile (31 S) inferred from 10 Be dating. Earth Planet. Sci. Lett. 277, 50–63. (doi:10.1016/j.epsl.2008.09.039)

[46] Saillard M, Riotte J, Regard V, Violette A, Hérail G, Audin L, Riquelme R 2012 Beach ridges U–Th dating in Tongoy bay and tectonic implications for a peninsula–bay system, Chile. J. South Amer. Earth Sci. 40, 77–84. (doi:10.1016/j.jsames.2012.09.001)

[47] Wolff M, Alarcón E 1993 Structure of a scallop *Argopecten purpuratus* (Lamarck, 1819)-dominated subtidal macro-invertebrate assemblage in northern Chile. J. Shellfish Res. 12, 295–304.

[48] Aldea C, Valdovinos C 2005 Moluscos del Intermareal Rocoso del Centro-Sur de Chile (36–38 S): Taxonomia y Clave de Identificacion (Rockyshore mollusks of the South-Central Chile (36–38 S): taxonomy and key of identification). Gayana 69, 364–396. (doi:10.4067/S0717-65382005000200014)

[49] Guzmán N, Saá S, Ortlieb L 1998 Catálogo descriptivo de los moluscos litorales (Gastropoda y Pelecypoda) de la zona de Antofagasta, 23 S (Chile). Estud. Oceanol. 17, 17–86.

[50] Marincovich L 1973 Intertidal mollusks of iquique, Chile. Los Angeles, CA: Natural History Museum.

[51] McLean JH 1984 Systematics of Fissurella in the Peruvian and Magellanic faunal provinces (Gastropoda: Prosobranchia). Los Angeles, CA: Natural History Museum of Los Angeles County.

[52] Servicio Nacional de Pesca, Medidas de Administración Pesquera. 2016 (cited 2016 June); See http://www.sernapesca.cl/.

[53] Kidwell SM 2007 Discordance between living and death assemblages as evidence for anthropogenic ecological change. Proc. Natl Acad. Sci. USA 104, 17 701–17 706. (doi:10.1073/pnas.0707194104) 10.1073/pnas.0707194104 PMC2077045 17965231

[54] Tomašových A, Kidwell SM 2011 Accounting for the effects of biological variability and temporal autocorrelation in assessing the preservation of species abundance. Paleobiology 37, 332–354. (doi:10.1666/09506.1)

[55] Oksanen J et al. 2013 Package ‘vegan’. *Community ecology package, version*. 2.

[56] R Core Team. 2015 R: a language and environment for statistical computing. Vienna, Austria (https://www.R-project.org/)

[57] Shannon C, Weaver W 1963 The measurement theory of communication. Urbana, IL: University of Illinois Press.

[58] Pielou EC 1966 The measurement of diversity in different types of biological collections. J. Theor. Biol. 13, 131–144. (doi:10.1016/0022-5193(66)90013-0)

[59] Chao A, Chazdon RL, Colwell RK, Shen T-J 2004 A new statistical approach for assessing similarity of species composition with incidence and abundance data. Ecol. Lett. 8, 148–159. (doi:10.1111/j.1461-0248.2004.00707.x)

[60] Kidwell SM 2001 Preservation of species abundance in marine death assemblages. Science 294, 1091–1094. (doi:10.1126/science.1064539) 11691990 10.1126/science.1064539

[61] Vavrek MJ 2011 Fossil: palaeoecological and palaeogeographical analysis tools. Palaeontol. Electron. 14, 1T.

[62] McGill BJ et al. 2007 Species abundance distributions: moving beyond single prediction theories to integration within an ecological framework. Ecol. Lett. 10, 995–1015. (doi:10.1111/j.1461-0248.2007.01094.x) 17845298 10.1111/j.1461-0248.2007.01094.x

[63] Prado P, Miranda M, Chalom A 2014 Package ‘sads’: Maximum likelihood models for species abundance distributions. See cran.r-project.org/web/packages/sads/index.html.

[64] O'Dea A, Shaffer ML, Doughty DR, Wake TA, Rodriguez FA 2014 Evidence of size-selective evolution in the fighting conch from prehistoric subsistence harvesting. Proc R Soc. B 281, 20140159 (doi:10.1098/rspb.2014.0159) 10.1098/rspb.2014.0159 PMC3973277 24648229

[65] Fenberg PB, Roy K 2008 Ecological and evolutionary consequences of size-selective harvesting: how much do we know? Mol. Ecol. 17, 209–220. (doi:10.1111/j.1365-294X.2007.03522.x) 17868288 10.1111/j.1365-294X.2007.03522.x

[66] Kosnik MA, Jablonski D, Lockwood R, Novack-Gottshall PM 2006 Quantifying molluscan body size in evolutionary and ecological analyses: maximizing the return on data-collection efforts. Palaios 21, 588–597. (doi:10.2110/palo.2006.p06-012r)

[67] Stotz W et al. 2006 Estudio reproductivo del recurso almeja en la IV Región. Informe final Proyecto FIP 46, 154.

[68] Webster JM, Davies PJ 2003 Coral variation in two deep drill cores: significance for the Pleistocene development of the Great Barrier Reef. Sediment. Geol. 159, 61–80. (doi:10.1016/S0037-0738(03)00095-2)

[69] DiMichele WA, Behrensmeyer AK, Olszewski TD, Labandeira CC, Pandolfi JM, Wing SL, Bobe R 2004 Long-term stasis in ecological assemblages: evidence from the fossil record. Annu. Rev. Ecol. Evol. Syst. 35, 285–322. (doi:10.1146/annurev.ecolsys.35.120202.110110)

[70] Pandolfi JM, Jackson JB 2006 Ecological persistence interrupted in Caribbean coral reefs. Ecol. Lett. 9, 818–826. (doi:10.1111/j.1461-0248.2006.00933.x) 16796572 10.1111/j.1461-0248.2006.00933.x

[71] Valentine JW, Jablonski D 1991 Biotic effects of sea level change: the Pleistocene test. J. Geophys. Res. 96, 6873–6878. (doi:10.1029/90JB00602)

[72] Lotze HK, Coll M, Magera AM, Ward-Paige C, Airoldi L 2011 Recovery of marine animal populations and ecosystems. Trends Ecol. Evol. 26, 595–605. (doi:10.1016/j.tree.2011.07.008) 21852017 10.1016/j.tree.2011.07.008

[73] Jones HP, Schmitz OJ 2009 Rapid recovery of damaged ecosystems. PLoS ONE 4, e5653 (doi:10.1371/journal.pone.0005653) 19471645 10.1371/journal.pone.0005653 PMC2680978

[74] Gardiner L 2001 Stability of Late Pleistocene reef mollusks from San Salvador Island, Bahamas. Palaios 16, 372–386. (doi:10.1669/0883-1351(2001)016<0372:SOLPRM>2.0.CO;2)

[75] Rivadeneira MM, Carmona ER 2010 A late Pleistocene macrobenthic assemblage in Caleta Patillos, northern Chile: paleoecological and paleobiogeographical interpretations. Andean Geol. 35, 163–173. (doi:10.5027/andgeoV35n1-a08)

[76] Carré M et al. 2012 Mid-Holocene mean climate in the south eastern Pacific and its influence on South America. Quat. Int. 253, 55–66. (doi:10.1016/j.quaint.2011.02.004)

[77] Lamy F, Hebbeln D, Röhl U, Wefer G 2001 Holocene rainfall variability in southern Chile: a marine record of latitudinal shifts of the Southern Westerlies. Earth Planet. Sci. Lett. 185, 369–382. (doi:10.1016/S0012-821X(00)00381-2)

[78] Maldonado A, Villagrán C 2002 Paleoenvironmental changes in the semiarid coast of Chile (∼ 32 S) during the last 6200 cal years inferred from a swamp–forest pollen record. Quat. Res. 58, 130–138. (doi:10.1006/qres.2002.2353)

[79] Maldonado A, Villagrán C 2006 Climate variability over the last 9900 cal yr BP from a swamp forest pollen record along the semiarid coast of Chile. Quat. Res. 66, 246–258. (doi:10.1016/j.yqres.2006.04.003)

[80] Dietl GP, Smith JA 2016 Live–dead analysis reveals long-term response of the estuarine bivalve community to water diversions along the Colorado River. Ecol. Eng. 106, 749–756. (doi:10.1016/j.ecoleng.2016.09.013)

[81] Lopez DA, Lopez BA, Gonzalez ML 2008 Shellfish culture in Chile. Int. J. Environ. Pollut. 33, 401–431. (doi:10.1504/IJEP.2008.02057)

[82] Oliva ME, Sánchez MF 2005 Metazoan parasites and commensals of the northern Chilean scallop *Argopecten purpuratus* (Lamarck, 1819) as tools for stock identification. Fish. Res. 71, 71–77. (doi:10.1016/j.fishres.2004.07.009)

[83] Sandin SA et al. 2008 Baselines and degradation of coral reefs in the Northern Line Islands. PLoS ONE 3, e1548 (doi:10.1371/journal.pone.0001548) 18301734 10.1371/journal.pone.0001548 PMC2244711

[84] Tyler CL, Kowalewski M 2017 Surrogate taxa and fossils as reliable proxies of spatial biodiversity patterns in marine benthic communities. Proc R. Soc. B 284, 20162839 (doi:10.1098/rspb.2016.2839) 10.1098/rspb.2016.2839 PMC5360931 28250189

[85] Pérez-Matus A et al. 2017 Temperate rocky subtidal reef community reveals human impacts across the entire food web. Mar. Ecol. Prog. Ser. 567, 1–16. (doi:10.3354/meps12057)

[86] Kidwell SM 2015 Biology in the Anthropocene: challenges and insights from young fossil records. Proc. Natl Acad. Sci. USA 112, 4922–4929. (doi:10.1073/pnas.1403660112) 25901315 10.1073/pnas.1403660112 PMC4413286

[87] Willis KJ, Araujo MB, Bennett KD, Figueroa-Rangel B, Froyd CA, Myers N 2007 How can a knowledge of the past help to conserve the future? Biodiversity conservation and the relevance of long-term ecological studies. Phil. Trans. R. Soc. B 362, 175–186. (doi:10.1098/rstb.2006.1977) 17255027 10.1098/rstb.2006.1977 PMC2311423

[88] Martinelli J, Soto L, Gonzalez J, Rivadeneira M 2017 Data from: Benthic communities under anthropogenic pressure show resilience across the Quaternary Dryad Digital Repository. (doi:10.5061/dryad.2g5f9) 10.1098/rsos.170796 PMC5627121 28989781

