# Supplementary material for: Benthic communities under anthropogenic pressure show resilience across the Quaternary
Source: R Soc Open Sci. 2017 Sep 20;4(9):170796. doi: 10.1098/rsos.170796 (PMC5627121; doi:10.1098/rsos.170796)
Supplement: Supplementary material for Martinelli et al. [file rsos170796supp1.docx]

Electronic Supplementary material

**Benthic communities under anthropogenic pressure show resilience across the Quaternary**

Julieta C. Martinelli ^1,2*^, Luis P. Soto^3^, Jorge González^2^ & Marcelo M. Rivadeneira^1,2^

^1^Laboratorio de Paleobiología, Centro de Estudios Avanzados en Zonas Áridas (CEAZA), Av. Bernardo Ossandón 877, CP. 1781681, Coquimbo, Chile ^2^Universidad Católica del Norte, Facultad de Ciencias del Mar, Departamento de Biología Marina, Coquimbo, Chile, ^3^Florida Museum of Natural History, University of Florida, Gainesville, Florida, USA

* Corresponding author: martinellijulieta@gmail.com

**FIGURES**

**Supplementary Figure 1.** Species relative abundance in (A) Pleistocene assemblages as a function of relative abundances in Holocene assemblages, and (B) Holocene assemblages as a function of relative abundances in Modern assemblages. The dashed line indicates a 1:1 relationship.

**Supplementary Figure 2.** Landings (in tons) for *Mulinia edulis* in Tongoy Bay over the last 22 years. Data from Servicio Nacional de Pesca (Sernapesca, 2016).

**TABLES**

**Supplementary Table 1.** Spearman rho for rank order correlations and Chao’s Jaccard similarity index for total assemblage, only ‘Exploited’ species and only ‘Non-exploited’ species respectively. Values in bold indicate significant correlations.

| Sample | Total assemblage | | Exploited | | Non-Exploited | |
| --- | --- | --- | --- | --- | --- | --- |
|  | Spearman’s rho | Chao’s J | Spearman’s rho | Chao’s J | Spearman’s rho | Chao’s J |
| LIVE-DA | **0.29** | 0.64 | **0.76** | 0.96 | 0.08 | 0.56 |
| LIVE-HOL | **0.34** | 0.87 | 0.44 | 0.67 | 0.25 | 0.76 |
| LIVE-LP | 0.16 | 0.99 | 0.20 | 0.84 | **0.44** | 0.97 |
| LIVE-MP | **0.37** | 0.90 | 0.43 | 0.58 | **0.36** | 0.96 |
| DA-HOL | **0.67** | 0.99 | **0.56** | 0.93 | **0.59** | 0.90 |
| DA-LP | **0.34** | 0.98 | 0.25 | 0.91 | 0.15 | 0.33 |
| DA-MP | **0.40** | 0.68 | 0.25 | 0.68 | 0.46 | 0.22 |
| HOL-LP | **0.70** | 0.96 | **0.77** | 0.98 | **0.37** | 0.59 |
| HOL-MP | **0.63** | 0.90 | **0.78** | 0.98 | **0.39** | 0.67 |
| LP-MP | **0.52** | 0.87 | **0.59** | 0.99 | 0.22 | 0.33 |

**Supplementary Table 2.** Maximum likelihood fit from the ‘radfit’ function for the (A) Modern (B) Holocene and (C) Pleistocene samples. Broken stick model has no free parameters so no actual fit occurs. Low AIC values are interpreted as providing support for a given model. *** indicates a level of significance < 0.0001

A) MODERN

| Model | Estimate | Std. Error | z value | Pr (z) | AIC |
| --- | --- | --- | --- | --- | --- |
| Geometric Series | 0.249 | 0.003 | 83.033 | 2.2 x 10^-6^ *** | 23367.37 |
| Broken stick | - | - | - | - | 27744.90 |
| Zipf | 1.625 | 0.014 | 117.12 | 2.2 x 10^-6^ *** | 21241.67 |

B) HOLOCENE

| Model | Estimate | Std. Error | z value | Pr (z) | AIC |
| --- | --- | --- | --- | --- | --- |
| Geometric Series | 0.339 | 0.004 | 88.429 | 2.2 x 10^-6^ *** | 19558.92 |
| Broken stick | - | - | - | - | 23514.77 |
| Zipf | 1.864 | 0.017 | 108.87 | 2.2 x 10^-6^ *** | 16686.08 |

C) PLEISTOCENE

| Model | Estimate | Std. Error | z value | Pr (z) | AIC |
| --- | --- | --- | --- | --- | --- |
| Geometric Series | 0.287 | 0.003 | 85.05 | 2.2 x 10^-6^ *** | 21605.85 |
| Broken stick | - | - | - | - | 24834.66 |
| Zipf | 1.635 | 0.015 | 109.08 | 2.2 x 10^-6^ *** | 19987.53 |

**Supplementary Table 3.** T-test results comparing size for the different time intervals for *Argopecten purpuratus* and *Mulinia edulis*. Significant p values are indicated in bold.

| Sample | *Argopecten purpuratus* | | *Mulinia edulis* | |
| --- | --- | --- | --- | --- |
|  | t | p value | t | p value |
| DA-HOL | -1.40 | 0.16 | 9.33 | **2.2x10^-16^** |
| DA-LP | -0.33 | 0.74 | 14.47 | **2.2x10^-16^** |
| DA-MP | 2.09 | **0.04** | -3.30 | **0.005** |
| HOL-LP | 1.69 | 0.09 | 8.46 | **2.2x10^-16^** |
| HOL-MP | 3.38 | **0.001** | -6.33 | **2.19x10^-05^** |
| LP-MP | 1.66 | 0.11 | -7.78 | **2.66x10^-06^** |

**DATASETS**

Six datasets are provided in ‘.csv’ format. The files contain the community composition data for the different times bins as follows:

- 'TB_MP.csv': Mid Pleistocene data from Tongoy Bay.

- 'TB_LP.csv': Late Pleistocene data from Tongoy Bay.

- 'TB_HOL.csv': Holocene data from Tongoy Bay.

- 'TB_DA.csv': Dead assemblage data from Tongoy Bay.

- 'TB_Live91.csv': Live collected data from Tongoy Bay, 1991.

- 'TB_Live12.csv': Live collected data from Tongoy Bay, 2012.

- ‘TB_size.csv’: Size data for *Mulinia* and *Argopecten*.

**R CODE**

An ‘.R’ file with the code used for analyses and figures is included under the following name:

- ‘Martinelli et al._PRSB.R’
